# Prevalence and related factors of Active and Healthy Ageing in Europe according to two models: Results from the Survey of Health, Ageing and Retirement in Europe (SHARE)

**DOI:** 10.1371/journal.pone.0206353

**Published:** 2018-10-29

**Authors:** Cristina Bosch-Farré, Josep Garre-Olmo, Anna Bonmatí-Tomàs, Maria Carme Malagón-Aguilera, Sandra Gelabert-Vilella, Concepció Fuentes-Pumarola, Dolors Juvinyà-Canal

**Affiliations:** 1 Health and Healthcare Research Group, University of Girona, Girona, Catalonia, Spain; 2 Nursing Department, University of Girona, Girona, Catalonia, Spain; 3 Girona Biomedical Research Institute (IDIBGI), Salt, Catalonia, Spain; 4 Healthcare Institute (IAS), Salt, Catalonia, Spain; 5 Department of Medical Sciences, University of Girona, Girona, Catalonia, Spain; IRCCS E. Medea, ITALY

## Abstract

**Background:**

Active and Healthy Ageing (AHA) is the process of optimizing opportunities related to health, participation, and safety in order to improve quality of life. The approach most often used to measure AHA is Rowe and Kahn’s Satisfactory Ageing model. Nonetheless, this model has limitations. One of the strategic objectives of the WHO Global Strategy and Action Plan (2016) is to improve Healthy Ageing measurement. Our objectives were to compare two models of assessing AHA and further compare the results by country and sociodemographic variables.

**Methods:**

This was a cross-sectional, observational analysis of a representative sample of the general population aged 50 years and older in Europe. The data analysed were obtained by the Study of Health, Ageing and Retirement in Europe (SHARE). The dependent variable was AHA and its dimensions, measured using the Rowe and Kahn AHA model (AHA-B) and the authors’ model based on the WHO definition (AHA-BPS). A descriptive analysis and multivariate models of binary logistical regression were developed.

**Results:**

The sample consisted of 52,641 participants (mean age 65.24 years [SD = 10.18; Range = 50–104], 53.2% women). Healthy Ageing prevalence in the AHA-B model was 23.5% (95%CI = 23.1%-23.9%). In the AHA-BPS model, this prevalence was 38.9%. In both models, significant variations were observed between countries, and were distributed along a north-western to south-eastern gradient. The sociodemographic variables associated with the absence of AHA were advanced age, female sex, death of spouse, low educational level, lack of employment, and low financial status. Comparing the two models, the strength of association between absence of AHA and advanced age (85 years and older) was four times greater in the AHA-B model.

**Conclusions:**

Our results showing differences between these two models provide evidence that the AHA-BPS model does not penalize older age and is more likely to characterize AHA from a health promotion perspective.

## Introduction

The ageing of the world’s population has significant consequences in areas such as the labour market, pensions, health provisions, housing and social services [1,2] that can be seen as threatening the level of social welfare provided for older people. However, the older population is an important part of a society’s economic, social, and cultural capital. In this regard, the World Health Organization (WHO) and other global organizations emphasize the promotion of active and healthy ageing (AHA) among their priorities [3–6]. According to the WHO Strategy and action plan for healthy ageing in Europe 2012–2020, AHA can contribute to making health and well-being systems sustainable, especially by encouraging older people to stay active, independent, and fully integrated into society [6].

Nonetheless, a uniform definition of AHA is still lacking, which gives rise to ambiguity in its operationalization, causing heterogeneity of results [7,8] and multiple models of analysis that emphasize either biomedical or psychosocial aspects [7–10]. Cosco et al. described 105 ways of operationalizing the AHA that produced a range of prevalence from 0.4% to 91.7% [7]. The more flexible the model, the higher the prevalence of AHA, and the inverse was true: models that applied more restrictive criteria showed a lower AHA prevalence.

One of the most widely used biomedical models of AHA in the scientific literature is the Successful Ageing Model developed by Rowe and Kahn [11]. It includes 3 domains: 1) low probability of disease and disability; 2) maintenance of high physical and cognitive functioning; 3) engagement in social and productive activities. Despite the incorporation of the social dimension, this model is considered predominantly biomedical because its definition of AHA requires a physical criterion of no disease or disability [12–14]. According to this model, the prevalence of AHA in European studies ranges from 1.6% to 21.1% [15]. The Rowe and Kahn model has been applied and adapted by numerous authors [10,13,15–18].

The biomedical AHA model has created several controversies [8]. Some authors believe the presence of disease and disability to be a limiting factor in categorizing AHA [12,13,19–21]. The model also does not consider certain dimensions, especially mental well-being [7,8,12,22], and subjective variables that contribute to AHA [10,22–26]. For this reason, WHO advocates for a multidimensional AHA construct with a positive and bio-psycho-social (BPS) perspective that defines AHA as the process of optimizing opportunities for health, participation, and security, with the aim of increasing healthy life expectancy and quality of life [6]. The proposals to measure AHA based on the WHO definition include the 6 Factor Model by Paúl et al. (personal factors, behaviour determinants, determinants of social environment; determinants of health and social services; determinants of physical environment; economic determinants), which the authors were unable to validate empirically in their study [27]; the Active Ageing Index (AAI) developed by the European Commission, which emphasizes social engagement and measures AHA at national and subnational levels based on general population variables rather than individual characteristics, and therefore provides only an overview of ageing by country [28]; and the European Innovation Partnership on Active Healthy Ageing (INAHA) within the framework of the Europe 2020 strategy, an effort to operationalize AHA with tools such as WHODAS 2.0 (the revised WHO Disability Assessment Schedule) and Health-Related Quality of Life (HRQoL) [29]. WHODAS 2.0 measures disability and does not take a positive ‘healthy ageing’ perspective.

Although many AHA models exist, few are focussed specifically on the definition proposed by WHO, respecting the intrinsic AHA concepts of that definition and adopting a holistic and positive perspective on health. According to the Global Strategy and Action Plan (2016), which urges the establishment of the required evidence, one area to be addressed is the improvement of AHA evaluation, monitoring, and research [30]. The AHA-BPS model allows a detailed analysis of ageing using a database that is representative of the European population, together with a longitudinal study design that permits the definition of baseline values from which to monitor the evolution of AHA status and the impact of interventions in favour of AHA. A strength of this approach is the possibility to validate the model and obtain an instrument to assess AHA at the individual level.

As the WHO report points out, the extent of the opportunities that arise from increasing longevity will be heavily dependent on one key factor: the health of these older populations. If people are experiencing these extra years in good health and live in a supportive environment, their ability to do the things they value will have few limits [30]. Therefore, a comprehensive response is urgently needed to promote healthy ageing. The lack of a consensus definition of AHA has been a great weakness for researchers [7]. The objective of this project was to develop an AHA model in accordance with the WHO principles, measure the prevalence of AHA in 14 European countries, and compare the findings with the results obtained using the biomedical model.

## Material and methods

### Design

This was an analytical observational cross-sectional study.

### Population and sample

**Fig 1:**
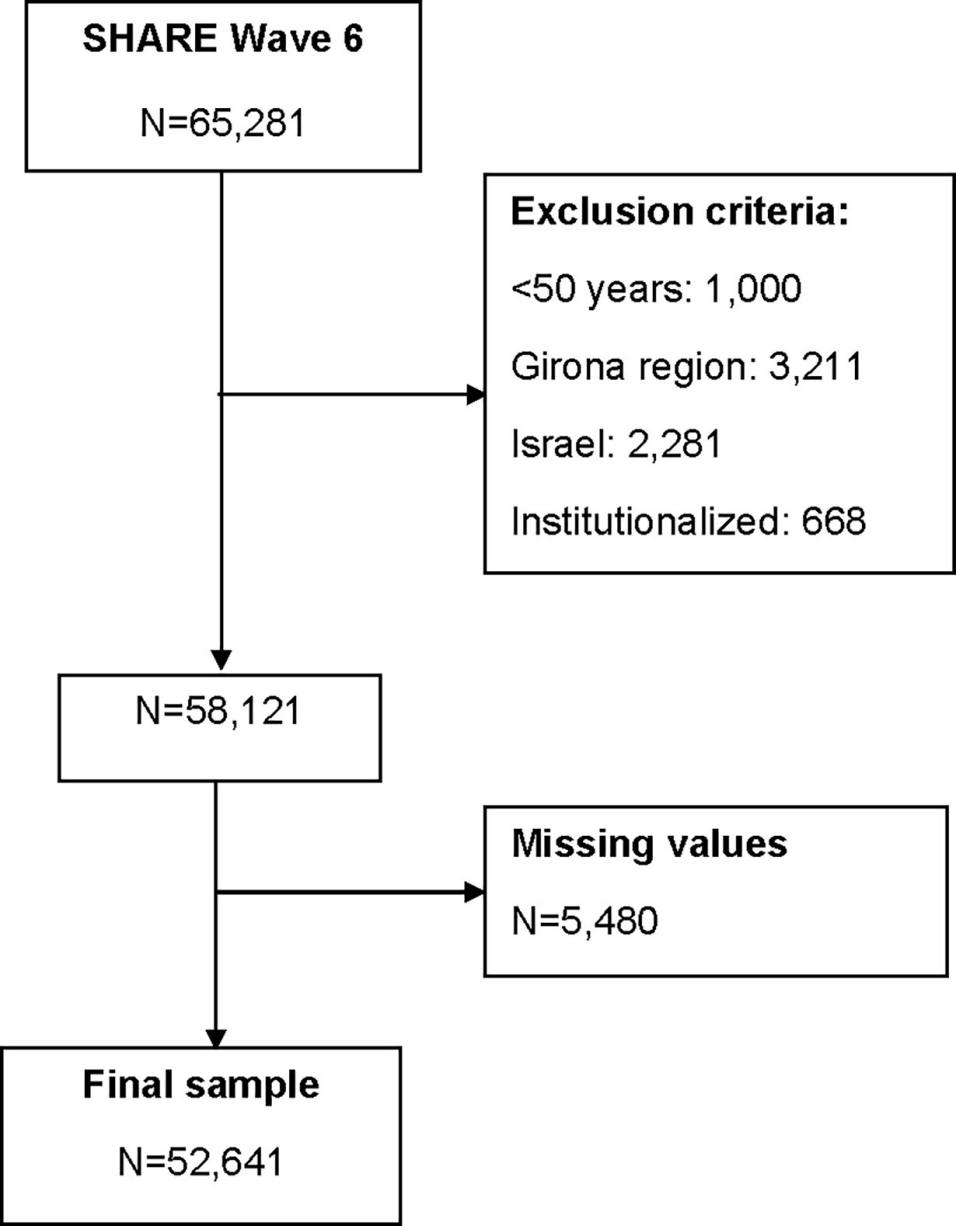
Flow chart.

The 2013 data analysed came from the fifth wave of the Study of Health, Ageing and Retirement in Europe (SHARE), which assessed the health, socioeconomic status, and social and family networks of community-dwelling people aged 50 and older in 15 countries (DOI: 10.6103/SHARE.w5.100) [31]. The exclusion criteria were residence in Israel or the region of Girona and missing data for any of the key variables in the AHA model (Fig 1). Despite the imputation strategies applied by SHARE researchers to minimize missing values, participants with at least one missing variable constituted 9.43% of eligible individuals (n = 5,480). Table in S1 Table shows the distribution of the final sample of 52,641 participants across 14 countries and the proportion of missing data per country.

### Procedure

SHARE is based on an in-home computer-aided personal interview (CAPI) that includes 22 modules collecting information on sociodemographic variables, physical and mental health, biomarkers, psychological and economic status, and social and family networks. The methodology, fieldwork procedures, and database have been previously described [32,33].

### Assessment of AHA

Initially, AHA was assessed with a biomedical model based on the Rowe and Kahn model (AHA-B) and the biopsychosocial AHA definition based on the WHO criteria (AHA-BPS). The variables to characterize the two models were extracted from 7 of the 22 SHARE modules: physical health, cognitive function, handgrip strength, mental health, social support, activities, and financial resources.

The **AHA-B model** was operationalized according to McLaughlin et al. [17], as modified by Hank according to the variables available to SHARE [15]. A person fulfils AHA criteria if 3 conditions are met: (a) no disease, no disability; (b) high cognitive and physical functioning; (c) active engagement in society. Each condition is operationalized with the following indicators [15]:

- **Absence of major diseases.** No doctor has ever told the respondents they have cancer, chronic lung disease, diabetes, heart disease, or stroke, or they had no scores of 4 or higher in the EURO-D depression scale [34, 35]. **Absence of disability**. The respondents reported no limitation in basic activities of daily living (ADL): walking indoors, dressing, bathing or showering, eating, getting into or out of bed, toileting.
- A **high level of cognitive function** was considered to be an above-average total score on all tests of mental orientation, memory, and mathematical calculations (maximum score, 29 points): correctly state the day of the week, date, month, and year (1 point for each); remember list of 10 words, immediate and delayed recall (1 point for each); complete 5 consecutive mathematical calculations (subtractions, 1 point per answer). **High level of physical function** was recorded if the person had difficulty with no more than one of the following six activities: climb a full flight of stairs, climb several flights of stairs, lift more than 5 kg, lean / squat / kneel, push large objects, walk 100 meters.
- **Social engagement** included paid work or volunteering in the previous month or caring for grandchildren at some time during the past 12 months. **Social support** was defined as living with a partner, providing economic or household help to relatives, friends, or neighbours, or engaging in a sport in the previous month.

The **AHA-BPS** model also assesses three dimensions, each using two indicators. The WHO definition includes physical, mental, and social well-being dimensions, operationalized as follows:

- **Physical well-being.** The two indicators were **non-frail or pre-frail** according to the SHARE-FI scale [36] and **good cognitive function**, defined as meeting the orientation criterion (AHA-B, above) and being above the 10^th^ percentile (p10), age-adjusted (age groups: 50–64, 65–74, 75–84, and 85 years and older) in at least 3 of 4 cognitive tests (immediate memory, delayed memory, mathematical calculation, verbal flow).
- **Mental well-being.** The two indicators were self-reported **satisfaction with life** (defined as 7 or higher on a 0–10 scale) and **no depressive symptoms** (defined as having 3 or fewer symptoms on the Euro-D scale) [34].
- **Social well-being.** The two indicators were **social participation,** defined as any paid work, caring for grandchildren, and social activities (sports, training, religion, politics, volunteering) and a satisfaction score of 7 or higher on a 0–10 scale in the social activity performed and **social support**, including social network (i.e., having given or received financial or household help) and family support. For people with children or a partner, family support was defined as living with a partner or child or living within 5km of a son or daughter and a score <5 (never or seldom feeling alone) on the Revised University of California-Los Angeles (R-UCLA) Loneliness Scale [37]. For those without a partner or children, only the subjective criterion (R-UCLA <5) was measured.

The variables drawn from the SHARE database are shown in Supplementary S2 Table (S2 Table. Coding of dependent and independent variables selected from the SHARE database).

### Independent variables

The study analysed seven sociodemographic variables: 1) age and age group (50–64, 65–74, 75–84, 85 years and older; 55 years and older; 65 years and older); 2) sex; 3) marital status (married or stable partner, single, divorced or separated, widowed); 4) educational level according to International Standard Classification of Education (ISCED-97), categorized as low (less than secondary education), moderate (completed secondary education) and high (some postsecondary education) [38]; 5) employment status (retired, actively employed or self-employed, and other, such as unemployment, homemaker, or disabled); 6) economic status related to perception of income adequacy (reaches the end of the month without difficulty [easily or more than easily], with some difficulty, or with great difficulty); and 7) country of residence (Austria, Belgium, Denmark, Estonia, France, Germany, The Netherlands, Italy, Luxembourg, Slovenia, Spain, Sweden, Switzerland, Czech Republic).

### Statistical analysis

The prevalence of AHA in both models was determined by relative frequencies with 95% confidence intervals (95%CI), stratified by age group and country. The prevalence of AHA in the two models was compared and, according to the different dimensions, stratified by sex, age group, and socioeconomic status. Differences in the prevalence of AHA dimensions were compared with Chi-square tests for age groups and geographical locations. To determine if the differences between the two models were statistically significant, the relative frequencies (Chi-square test) and the odds ratio were calculated. Weighted statistical samples were used to minimize the potential for selection bias in different countries [33]. Multivariate logistic regression was used to analyse the strength of the association in both models between AHA and the sociodemographic variables. Results are expressed as absolute numbers and percentages, means, standard deviations (SD), odds ratios (OR), and 95% confidence intervals (CI). Statistical tests were considered to be significant with a two-tailed p value < 0.05. The data were analysed using SPSS.19.

### Ethics statement

Use of the SHARE data (5th wave) was reviewed and approved by the Ethics Committee of the Max Planck Society for the Progress of Science. SHARE-ERIC's activities related to human subjects research are guided by international research ethics principles such as the Respect Code of Practice for Socio-Economic Research and the Declaration of Helsinki.

## Results

Mean age was 65.24 years (SD = 10.18; Range = 50–104); 52.6% of participants were aged 50–64 years, followed by 65–74 years (26.8%), 75–84 years (16.1%), and 85 years and older (4.5%). Women were 53.2% of the sample. Most participants (72.1%) were married, and 21.8% had a high educational level, 38.3% a moderate level, and 39.9% a low level. While 48.7% of participants were retired, 33.3% were actively employed. When asked about making ends meet, or “getting to the end of the month”, 33.0% reported having economic difficulty (23.3% some difficulty and 9.7% great difficulty). In supplementary information, Table in S3 Table shows a summary of participant characteristics, by country.

**Fig 2:**
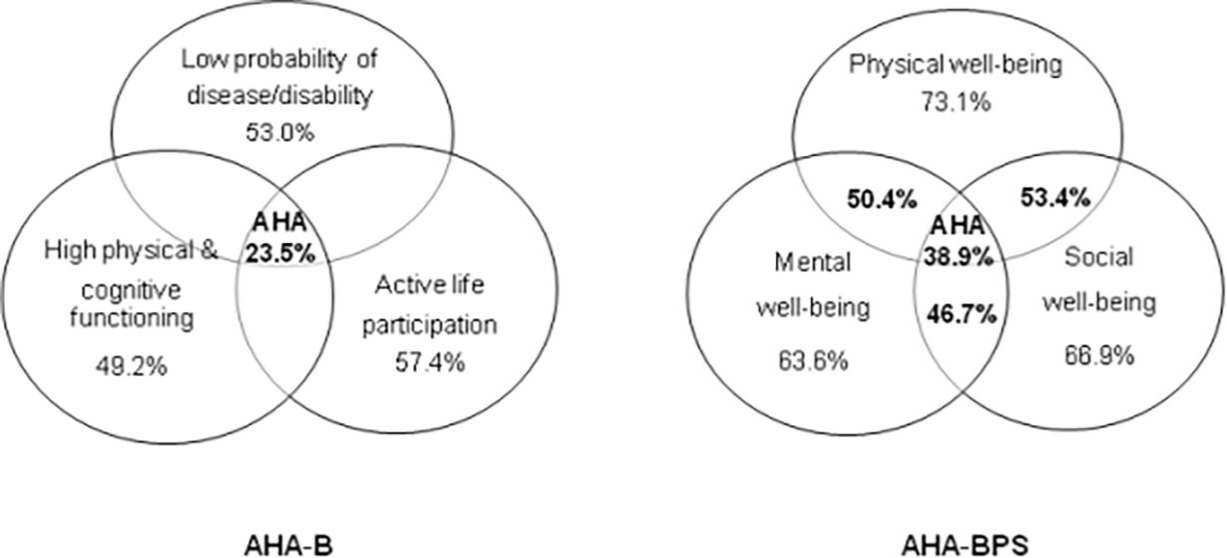
Venn diagram of the AHA‐B and AHA‐BPS models.

The overall prevalence of AHA was 23.5% (95%CI = 23.1%-23.9%) in the AHA-B model and 38.9% (95%CI = 38.5%-39.3%) in the AHA-BPS model. Fig 2 shows the percentages of participants that fulfilled each dimension and, for the AHA-BPS model, the results for the combination of each pair of dimensions. The criteria that were least often met were physical and cognitive functioning in the AHA-B model and mental well-being in the AHA-BPS model.

When the models were compared, the degree of correlation was low (Kappa Index = 0.475). In the distribution of relative frequencies, 19.6% of participants reported AHA according to both the AHA-B model and the AHA-BPS model; in contrast, 57.2% of people who did not meet AHA criteria according to the AHA-B model also did not meet them using the AHA-BPS model. According to the AHA-BPS model, 21,689 individuals reported AHA, 8,428 (63.55%) more than the 13,261 identified by the AHA-B model (Table 1).

**Table 1:** Comparison of AHA-B and AHA-BPS models.

	**AHA-BPS**		
AHA-B	YES	NO	TOTAL
**YES**	11,121	2,140	13,261
**NO**	10,568	28,812	39,380
**TOTAL**	21,689	30,952	52,641

Tables 2 and 3 show the prevalence of each component of the operational definition of the AHA models, stratified by criteria and by age groups. In both models, the older the age group, the less likely were they to report AHA. This decline was more pronounced after 75 years of age; in the AHA-B model, 34.3% of people aged 50–64 years had AHA, compared to 1.3% of people aged 85 and older. Overall, the criterion that most inhibited AHA was disease or disability (45.0%). In those aged 75 and older, two criteria were most often not met: social participation and high cognition. In the AHA-BPS model, which does not include the disease criterion, the dimension that decreased most with age was social welfare, while the dimension that remained more stable was mental well-being.

**Table 2:** Prevalence of AHA in AHA-B model, by dimensions and age groups *.

	Age groups (%) **				50+	55+	65+	Hank^1^	Mc-Laughlin^2^
	50–64	65–74	75–84	+85	(%)	(%)	(%)	(%)	(%)
**D1. Low probability of disease/disability**	**61.0**	**50.9**	**37.3**	**29.1**	**53.0**	**50.9**	**44.2**		
No disease	62.4	52.2	40.7	35.5	55.0	52.9	46.7	42.6	37.0
No disability	94.6	92.3	84.7	66.1	91.1	90.3	87.3	83.7	82.1
**D2. High physical and cognitive functioning**	**62.4**	**46.1**	**22.9**	**8.9**	**49.2**	**45.9**	**40.7**		
High cognitive functioning	69.7	54.9	33.5	17.3	57.5	54.4	52.3	48.5	57.8
High physical functioning	87.2	79.2	59.9	43.1	78.7	76.7	69.2	57.3	49.0
**D3. Active life participation**	**73.1**	**52.8**	**27.6**	**9.8**	**57.4**	**54.2**	**40.1**	**27.1**	**49.7**
Social participation	83.0	60.0	33.5	12.4	65.7	62.0	46.5		
Social support	86.7	84.4	76.9	75.3	84.0	83.4	81.0		
**TOTAL AHA**	**34.3**	**17.0**	**5.2**	**1.3**	**23.5**	**20.5**	**13.1**	**8.5**	**10.9**

Note

*Percentages are weighted.

** p<0.001 for age groups

^1^ Data from Hank (2010) based on SHARE data for participants aged 65 and older (Waves 1 and 2, 2004–2007).

^2^ Data from McLaughlin et al. (2010) based on U.S. Health and Retirement Study in the population aged 65 and older.

**Table 3:** Prevalence of AHA in AHA-BPS model, by dimensions and age groups *.

	Age groups (%) **				50+	55+	65+
	50–64	65–74	75–84	85+	(%)	(%)	(%)
**D1. Physical well-being**	**77.5**	**74.7**	**63.9**	**44.8**	**73.1**	**71.9**	**68.2**
No frailty	97.5	95.0	85.6	71.1	93.8	93.1	89.6
High cognition	78.8	77.6	70.1	60.2	76.3	75.4	73.4
**D2. Mental well-being**	**65.2**	**66.7**	**56.8**	**51.0**	**63.6**	**63.3**	**61.9**
Satisfied with life	79.8	81.4	75.1	74.7	79.2	79.1	78.6
No depression	75.3	76.4	68.2	61.4	73.8	73.8	72.2
**D3. Social well-being**	**80.3**	**63.8**	**40.4**	**23.5**	**66.9**	**64.2**	**52.0**
Social participation	85.8	68.6	44.4	26.2	71.9	69.0	56.4
Social support	92.1	90.0	87.5	84.5	90.5	90.1	88.6
**TOTAL AHA**	**46.9**	**38.0**	**22.3**	**8.8**	**38.9**	**36.9**	**29.9**

Note

*Percentages are weighted.

** p<0.001 for age groups

**Fig 3:**
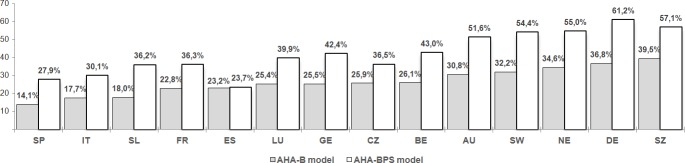
Prevalence of AHA in both models, by country *.

Stratified by country, AHA prevalence ranged from 14.1% in Spain to 39.5% in Switzerland in the AHA-B model. In contrast, using the AHA-BPS model, the prevalence ranged from 23.7% in Estonia to 61.2% in Denmark. In both models, the countries with the highest AHA prevalence were located in northern and western Europe. In the AHA-BPS model, more than half of the northern population reported AHA, compared to approximately a quarter of the participants in Estonia and Spain (23.7% and 27.9%, respectively). (Fig 3)

Analysed by country, less than half of the participants (46.6%) in Estonia met the criteria of dimension 1 (low probability of disease and disability) in the AHA-B model, compared to 67.9% in Switzerland. On dimension 2 (high cognitive and physical function), the lowest percentage (31.2%) was found in Spain, in contrast to Switzerland, where 70.9% of participants met the criteria. Likewise, less than half of the Spanish participants (44.7%) met the criteria for dimension 3 (participation in society), compared to 75% in Denmark. It should be noted that Spain scored low (55.5%) on the physical well-being dimension in the AHA-BPS model because it includes cognitive function and the Spanish participants again scored lower on that criterion in the AHA-B model (57.9%). Table 4 summarizes the AHA results for each model in each country, grouped by European geographic region according to Eurovoc and defined as follows: ‘northern’ (Denmark, Sweden, and Estonia), ‘eastern’ (Czech Republic and Slovenia), ‘southern’ (Italy and Spain); and ‘western’ (Austria, Belgium, France, Germany, Luxembourg, Holland / The Netherlands, and Switzerland) [39]. In the northern region, 8 out of 10 people had high cognitive function. Regarding mental well-being, the differences between countries were mainly based on life satisfaction. The countries of southern and eastern Europe scored lowest in the social welfare dimension, mainly due to the social participation criterion. In S4 Table shows a summary of AHA prevalence in both models, by country.

**Table 4:** Prevalence of AHA in both models, by the geographical location of Europe *.

	NORTH-WESTERN**	SOUTH-EASTERN**	NORTHERN^1^**	WESTERN^2^**	SOUTHERN^3^**	EASTERN^4^**	50+
(n)	38,540	14,101	12,880	25,660	6,542	7,559	52,641
**AHA-B MODEL**							
**D1. Low probability of disease / disability**	**52.7**	**53.7**	**58.4**	**52.2**	**53.8**	**52.5**	**53,0**
No disease	54.7	55.4	60.2	54.3	55.5	54.6	55,0
No disability	90.9	91.6	93.3	90.7	91.7	91.1	91.1
**D2. High physical & cognitive functioning**	**54.9**	**38.3**	**58.7**	**54.5**	**36.6**	**52.5**	**49.2**
High cognitive function	64.7	43.6	65.7	64.6	41.2	62.8	57.5
High physical function	79.8	76.5	85.5	79.3	76.5	76.5	78.7
**D3. Active participation**	**62.1**	**48.4**	**70.1**	**61.4**	**47.5**	**55.2**	**57.4**
Social participation	69.7	57.8	75,0	69.2	57.2	63.4	65.7
Social support	86.4	79.2	91.9	86,0	78.7	83.1	84,0
**TOTAL AHA-B**	**26.7**	**17.2**	**33.1**	**26.2**	**16.3**	**24.5**	**23.5**
**AHA-BPS MODEL**							
**D1. Physical well-being**	**77.9**	**63.6**	**80.9**	**77.7**	**61.8**	**78,0**	**73.1**
No frailty	95,0	91.3	96.5	94.9	90.9	94.4	93.8
High cognition	81.1	66.9	83.4	80.9	65.2	81.3	76.3
**D2. Mental well-being**	**65.1**	**60.8**	**74.8**	**64.3**	**61,0**	**59,0**	**63.6**
Satisfaction with life	80.5	76.8	88,0	79.9	77.6	70.1	79.2
No depression	75,0	71.4	80.9	74.6	70.9	75.8	73.8
**D3. Social well-being**	**71.8**	**57.5**	**80.2**	**71.1**	**56.8**	**63.2**	**66.9**
Social participation	76.4	63,0	83.5	75.8	62.2	69.1	71.9
Social support	92,0	87.5	94.7	91.7	87.5	87.7	90.5
**TOTAL AHA-BPS**	**43.4**	**30,0**	**45.5**	**42.4**	**29.3**	**36.4**	**38.9**

Note

*Percentages are weighted.

** p<0.001 for age groups

^1^ Northern: Denmark, Sweden, Estonia

^2^ Western: Austria, Belgium, France, Germany, Luxembourg, Holland /The Netherlands, Switzerland

^3^ Southern: Italy, Spain

^4^ Eastern: Czech Republic, Slovenia

Table 5 presents the results for participants who met AHA criteria in each model, stratified by sociodemographic variables. In both models, the prevalence of AHA was greater among younger participants, men, and those with more education, some employment, and without economic difficulty getting to the end of the month. In both models, those who were widowed had the lowest AHA prevalence.

**Table 5:** Percentage of participants with AHA, stratified by model and sociodemographic variables *.

		AHA-B		AHA-BPS	
Variable	n	%	(n)	%	(n)
**Age**					
50–64	24,265	39.2	(9.081)	56.8	(12,029)
65–74	17,051	14.0	(3.552)	33.3	(7,141)
75–84	9,172	4.9	(590)	21.9	(2,263)
≥85	2,153	1.1	(38)	10.3	(256)
**Sex**					
Men	23,612	26.9	(6.074)	49.0	(10,613)
Women	29,029	20.7	(7.187)	34.9	(11,076)
**Marital status**					
Married	37,974	25.6	(10.422)	45.3	(17,177)
Single	2,971	28.3	(708)	43.2	(1,016)
Divorced/Separated	4,907	23.4	(1.365)	34.1	(1,876)
Widowed	6,789	9.5	(766)	22.7	(1,620)
**Educational level**					
Low	19,022	13.2	(2.470)	29.0	(5,174)
Moderate	20,929	25.8	(5.721)	44.4	(9,198)
High	12,690	35.2	(5.070)	54.4	(7,317)
**Employment status**					
Retired	30,248	11.4	(4.865)	28.7	(10,576)
Employed	14,928	53.1	(7.377)	74.0	(9,273)
Other	7,465	13.3	(1.019)	26.2	(1,840)
**Economic status**^**1**^					
No difficulty	36,702	27.3	(10.952)	48.0	(18,068)
With difficulty	15,939	14.7	(2.309)	25.6	(3,621)

Note

*Percentages are weighted. Sample numbers (n) are not weighted.

^1^ Perception of income adequacy (reaches the end of the month with or without difficulty)

Table 6 includes odds ratios (OR) to show the strength of the association between a lack of AHA in each model and the main demographic subgroups. Using the AHA-B model, people aged 85 years and older were less likely to report AHA (OR = 15.271, 95%CI: 15.151–15.393) than those aged 75–84 years (OR = 3.843, 95%CI: 3.834–3.352), and those who were not working (OR = 3.459, 95%CI: 3.454–3.463) or had difficulty getting to the end of the month economically (OR = 2.149, 95%CI: 2.146–2.151). Using the AHA-BPS model, the corresponding subgroups were those aged 85 years and older (OR = 3.783, 95%CI: 3.771–3.796), not working (OR = 3.205, 95%CI: 3.202–3.209), and having economic difficulty (OR = 2.633, 95%CI: 2.630–2.635). Comparing the absence of AHA between the two models, the strength of its association with age (85 years and older) was four times greater in the AHA-B model, while economic difficulty was more strongly associated in the AHA-BPS model.

**Table 6:** Multivariate logistical regression of sociodemographic variables, by models *.

	AHA-B			AHA-BPS		
		95% CI			95% CI	
Variables	OR	Lower	Upper	OR	Lower	Upper
**Age**						
50–64	1	-	-	1	-	-
65–74	1.205	1.204	1.207	0.738	0.737	0.738
75–84	3.843	3.834	3.852	1.397	1.395	1.399
≥85	15.271	15.151	15.393	3.783	3.771	3.796
**Sex**						
Men	1	-	-	1	-	-
Women	1.019	1.018	1.020	1.282	1.281	1.283
**Marital status**						
Married or stable partner	1	-	-	1	-	-
Single, divorced, widowed	1.185	1.183	1.186	1.456	1.455	1.458
**Educational level**						
Moderate or high	1	-	-	1	-	-
Low	1.970	1.968	1.973	1.773	1.771	1.774
**Employment status**						
Active	1	-	-	1	-	-
Not active	3.459	3.454	3.463	3.205	3.202	3.209
**Economic status**^**1**^						
No difficulty	1	-	-	1	-	-
With difficulty	2.149	2.146	2.151	2.633	2.630	2.635

Note

*Percentages are weighted. Sample numbers (n) are not weighted.

^1^Perception of income adequacy (reaches the end of the month with or without difficulty)

## Discussion

This study compared two models of assessing AHA and further compared the results by country and by sociodemographic variables. The prevalence of AHA varied (15.4%) according to the operational definition used by each model [40,41], being 23.5% according to AHA-B, the more traditional biomedical model, and 38.9% according to the AHA-BPS model, based on the WHO criteria. The AHA-B model considers the presence of disease and disability a limiting factor in the achievement of AHA, and indeed 45% of the sample was excluded from the possibility of AHA in that analysis due to the presence of at least one of the six chronic diseases incorporated into the AHA-B construct. Other authors who use biomedical models have excluded nearly half of the sample due to illness [10,16,21], confirming disease and disability as the main factors limiting the categorization of AHA from a biomedical perspective [17, 20,42].

Authors who measure AHA with psychosocial or subjective dimensions have obtained a higher AHA prevalence, compared to the biomedical approach [13,21,43,44]. Similarly, people who self-report AHA may have chronic diseases [10,25,45,46]. Specifically, Parslow et al. found that those with the highest AHA scores also reported a mean of two chronic diseases [47]. Public health policies must be designed to maximize AHA among older people so that they are able to do what they want to do regardless of having a chronic disease; therefore, models of ageing should focus on function rather than disease or disability [48]. The AHA-BPS model does not include freedom from disease in its construct; rather, it measures frailty, using the SHARE-FI scale, which gives greater flexibility to AHA measurement and allows an exploration of the individual’s level of vulnerability, independent of any disease. In terms of *physical* health, frailty could be considered the opposite of AHA [8,49,50].

A number of studies confirm that the more flexible the AHA definition used, the more extensive is the AHA prevalence observed [12,13,19,41]. The more flexible models are more useful for public health purposes [12] because it is easier to identify areas of deficiency in multidimensional models, allowing more targeted actions in response [7]. Inui highlights the importance of applying an AHA construct with a holistic, non-reductionist perspective [51]. Nonetheless, some authors have concluded that when the definition incorporates more domains it becomes more restrictive and AHA prevalence is lower [42]. Based on our results, we concluded that the level of restriction in each domain has more impact than the number of domains considered and that the AHA-BPS model is less restrictive than the AHA-B model. Therefore, the AHA-BPS model permits the identification of vulnerable older populations requiring interventions designed to improve their health status, while the AHA-B model is less useful in public health planning because it identifies groups with better physical health, a smaller population (8,428 fewer individuals than were identified by AHA-BPS). Moreover, the AHA-BPS model contains all three dimensions included in the WHO definition of AHA (physical, mental, and social well-being), which have also been present in most other AHA models [7,9,52] and AHA qualitative studies [44,53].

Although mental well-being has been identified as a key factor in the positive perception of ageing [54], the AHA-B model considers it only as a criterion (specifically, a diagnosis of depression) in the calculation of the physical dimension that can exclude individuals from achieving AHA. Phelan and Anderson criticized this lack of a mental health dimension in the Rowe and Kahn construct [46]. In the AHA-BPS model, it is measured as a separate dimension. Psychological and social dimensions of ageing may not be positively associated with physical changes throughout life [45]. In addition, individuals may have mechanisms of compensation, resilience, and/or adaptation that help them adjust to the physical decline that is inherent in ageing and allow for AHA despite physical limitations and disease [13,45]. The person who is able to adapt to limitations and compensate for them by maximizing benefits and minimizing losses is better able to achieve “healthy ageing”: an optimal level of well-being and quality of life [45,55,56]. Therefore, psychosocial changes largely explain how old age can be a period of highly subjective well-being [57]. The findings in these earlier studies are in line with our results for the AHA-BPS model, where the most stable dimension over time was mental well-being, compared to other dimensions, which decreased with age. Perales et al. also found that the age gradient occurs in various AHA models, except in psychosocial AHA models that value subjective aspects of well-being [58]. This phenomenon is related to the so-called "subjective well-being paradox", according to which older people are not the least happy population despite having more health problems [59,60].

The AHA-BPS model showed 9.5% greater likelihood of meeting the criteria for social well-being, compared to the biomedical AHA-B model. Our AHA-B data also showed a progressive decrease in performance as age increased, a contrast with the findings by Weir et al. in a Canadian population, where the social domain varied little or not at all with age in their analysis (using the Rowe and Kahn model) [20]. In our study, social well-being was the dimension that decreased most with age in the AHA-BPS model. As other authors have observed, old age leads to considerable changes beyond biological decline, such as changes in social function and status and the need to face the loss of close relationships [3]. Loneliness is one of the social network variables most strongly associated with health [61]. The AHA-BPS model includes subjective measures. Other studies that measured psychosocial or subjective dimensions show high AHA prevalence compared to biomedical models [12,19–21].

Comparing AHA prevalence by country and model, according to the AHA-B model the population aged 65 years and older scored 4.6% higher in our study than was reported by Hank in 2010 [15]. In the Hank study, Denmark had the highest AHA prevalence, followed by The Netherlands, Sweden, and Switzerland, with the lowest prevalence in Mediterranean and eastern European countries. The comparison must take into consideration Hank's analysis of survey data from Waves 1 and 2 (our study used Wave 5 data) and differences in the countries studied (Poland, Greece, and Ireland did not participate in Wave 5, but it did include Luxembourg, Estonia, and Slovenia). In addition, Hank includes data from Israel. Other studies that have applied the AHA-B model in people aged 65 and older in the United States also obtained similar prevalence data, such as 18.8% in 2002 [10] or a lower prevalence in 2010 [17]. Comparing AHA prevalence by country, the results are consistent with Hank's north-western to south-eastern (Mediterranean and eastern Europe) gradient: Denmark, The Netherlands, Sweden, and Switzerland had the highest AHA prevalence and Spain had the lowest prevalence, with Italy in the penultimate place [15]. The analysis by age groups followed the same gradient, as in other studies that emphasized physical well-being measures such as disability [62]. The overall AHA prevalence according to the AHA-BPS model was 36.9% in people 55 and older, comparable to AAI data for the population aged 55 and older in 27 European countries (not including Switzerland). In 2012 data, the best AHA scores were obtained in Sweden and Denmark, while Estonia was ranked 16th of 27 countries, higher than Spain although both scores were very similar (33.1 and 32.5, respectively) [63]. In the AHA-BPS model, Denmark had the highest AHA prevalence, well ahead of fourth-place Sweden, and Estonia was in last place while Spain occupied the penultimate place. In 2014 AAI data analysed for 28 countries, Estonia was 10th while Spain remained in 17th place; Sweden and Denmark continued to lead with the best AHA scores and the lowest scores went to Poland and last-place Greece [64].

In general, both of these models showed a geographic pattern similar to that of the AHA-B model in earlier studies, in which the highest scores are observed in northern countries and western Europe, but with great variability in the order of countries listed [64]. Results reported by Perales et al. and Sowa et al. follow a similar geographic gradient [58,65]. Measuring mental well-being with a subjective variable, "satisfaction with life", affected the distribution of AHA by country. The countries of southern and eastern Europe that scored lower in this variable were below the global mean in the AHA-BPS model, while some of them were above the mean according to the AHA-B model. Other researchers corroborate these levels of satisfaction with life observed in northern and south-eastern Europe [66]. In addition, both models (AHA-B and AHA-BPS) found less social participation in the countries of southern and eastern Europe. Similarly, Litwin notes that non-Mediterranean men participate in more social activities, and do so more frequently, than do Mediterranean men [67]. Within the social network criterion, if economic or household help received from outside the home is calculated using the AHA-BPS model, the countries with the lowest scores on social networks are those of southern and western Europe, and Spain scored last. These data coincide with a study showing a great difference between northern and southern Europe. In the northern countries, older people who live alone receive more social assistance and institutional support, whereas in the Mediterranean countries, the support is provided by the family [68].

In relation to the sociodemographic variables associated with AHA, they were the same in both models: being male, younger, better educated, actively employed, and not having economic difficulty reaching the end of the month. Regarding AHA prevalence by age group, older age was associated with less AHA. This difference was greater in the AHA-B model, especially with respect to those aged 50–64 compared to the group aged 65–74, where the prevalence decreased almost 50% with older age. In the AHA-BPS model, AHA decreased more progressively with age. This age gradient has also been shown in previous studies [15–17,69,70]. A lower percentage of women reported AHA in both models, although the difference was greater in the AHA-BPS model, with men scoring 14.1 percentage points higher. This was mainly due to the AHA-BPS model’s inclusion of mental well-being variables, where women scored 13.2 percentage points lower. These results coincide with other authors who confirmed a higher percentage of AHA in men [15–17,71] and Bowling reported this difference both in the biomedical model and when the participants were asked for their self-perceptions about ageing [72]. Similarly, in two AAI datasets separated by sex, women generally had lower scores, regardless of differences between countries [63]. However, other authors have found little difference in AHA by sex [9,12,25,58] or greater AHA among women [8]. In most studies, a better socio-economic level was associated with a higher prevalence of AHA [15–17,19,69]. In the AHA-BPS model, more married people had AHA, compared to those who were widowed, divorced, or single, in concordance with other authors [23,58,70]. In both models we studied, the most significant difference related to marital status was to be widowed, although some authors have reported no differences according to marital status [23,69]. With respect to educational level, more education was associated with greater AHA in both models. The difference was greatest between the groups with the lowest and highest educational levels, coinciding with previous reports [16,58,70,73]. Having economic difficulty reaching the end of the month was associated with less AHA, more markedly in the AHA-B model. The AAI was also positively correlated with a country’s GDP per capita [64]. The OR for AHA was similar in both models for all sociodemographic variables except age: in the AHA-B model, people aged 85 and older were 15 times more likely to have AHA than people 50–64 years old; the difference was less than four times as likely in the AHA-BPS model. This finding provides greater evidence that the AHA-BPS model does not penalize older age (and the increased morbidity inherent to old age) and is more likely to characterize AHA from a health promotion perspective.

The main limitation of our work was that cross-sectional design precludes establishing causality relationships between the constructs or validating the predictive capacity of the AHA-BPS model. However, the potential exists for longitudinal analysis using Wave 6 SHARE data. The strength of this research is the comparison of two models of AHA assessment in a representative sample of 52,641 people from 14 European countries.

We concluded that the AHA-BPS model is more inclusive, allows measurement of AHA from a positive health perspective (without penalising for disease), and better identifies the population for which AHA promotion policies should be established. The contribution of this model is very important, in view of the global challenge that population ageing presents, because it applies the WHO criteria. Moreover, according to the Global Strategy and Action Plan (2016), our results reflect improved AHA measurement, monitoring, and research systems using this approach. The Global Strategy and Action Plan (2016) that was approved at the 69th World Assembly urges the establishment of the required evidence and partnerships necessary to support a Decade of Healthy Ageing from 2020 to 2030. One of the 10 mid-term progress indicators related to the action plan objectives is “Number of countries with cross-sectional, nationally representative, anonymous, individual-level data collected since 2010 on older adults and their health status and needs in the public domain” [30]. Analysis of the SHARE data on ageing and health offers a superb opportunity to contribute to the evidence needed to generate actions that will promote healthy ageing and health equity. The AHA-BPS model allows a detailed analysis of ageing in Europe and defines a baseline from which to monitor the evolution of the situation and the impact of the various interventions in favour of AHA [29]. Rowe recently affirmed that successful population ageing depends on how world societies adapt to the ageing phenomenon and invited others to learn from the European experience as an older continent [74].

As SHARE is a longitudinal study, future lines of research can be designed to validate the predictive value of the AHA-BPS model and to monitor and characterize the ageing of individuals, beginning at 50 years of age in some cases. Future research is needed to identify key public health actions carried out in the countries with the best AHA prevalence and to study the details of systems supporting health and well-being in greater depth, in order to find the strong points that have a direct impact on AHA and therefore contribute to improving the health and quality of life of older people.

## Supporting information

S1 Table Number of participants and missing values, by country. (DOCX)

S2 Table Coding of dependent and independent variables selected from the SHARE Database. (DOCX)

S3 Table Participant characteristics by country. (DOCX)

S4 Table Prevalence of AHA in both models, by country. (DOCX)
