# Supplementary material for: Prevalence and related factors of Active and Healthy Ageing in Europe according to two models: Results from the Survey of Health, Ageing and Retirement in Europe (SHARE)
Source: PLoS One. 2018 Oct 29;13(10):e0206353. doi: 10.1371/journal.pone.0206353 (PMC6205806; doi:10.1371/journal.pone.0206353)
Supplement: S1 Table — (DOCX) [file pone.0206353.s001.docx]

**S 1 Table. Number of participants and missing values, by country.**

|  | **Sample** | **Missing values** | **Final sample** | **Missing values (%)** |
| --- | --- | --- | --- | --- |
| Austria | 4,137 | 601 | 3,536 | 14.53 |
| Belgium | 5,419 | 385 | 5,034 | 7.10 |
| Czech Republic | 5,521 | 603 | 4,918 | 10.92 |
| Denmark | 4,000 | 139 | 3,861 | 3.48 |
| Estonia | 5,659 | 904 | 4,755 | 15.97 |
| France | 4,333 | 332 | 4,001 | 7.66 |
| Germany | 5,505 | 378 | 5,127 | 6.87 |
| Italy | 4,602 | 710 | 3,892 | 15.43 |
| Luxembourg | 1,552 | 129 | 1,423 | 8.31 |
| Netherlands | 4,023 | 241 | 3,782 | 5.99 |
| Slovenia | 2,910 | 269 | 2,641 | 9.24 |
| Spain | 3,081 | 431 | 2,650 | 13.99 |
| Sweden | 4,449 | 185 | 4,264 | 4.16 |
| Switzerland | 2,930 | 173 | 2,757 | 5.90 |
| **TOTAL** | **58,121** | **5,480** | **52,641** | **9.43** |
