# Supplementary material for: Prevalence and related factors of Active and Healthy Ageing in Europe according to two models: Results from the Survey of Health, Ageing and Retirement in Europe (SHARE)
Source: PLoS One. 2018 Oct 29;13(10):e0206353. doi: 10.1371/journal.pone.0206353 (PMC6205806; doi:10.1371/journal.pone.0206353)
Supplement: S2 Table — (DOCX) [file pone.0206353.s002.docx]

**S 2 Table. Coding of dependent and independent variables selected from the SHARE Database.**

| **VARIABLE NAME** | **SHARE CODE** |
| --- | --- |
| **AHA-B** | |
| No disease | ph006d_, mh002_, mh017_, mh014_, mh015_ |
| No disability | ph049_ |
| High cognitive functioning | cf003_-cf006_, cf007_, cf105_-cf107_, cf012_-cf015_, cf109_ - cf 116_ |
| High physical functioning | ph048_ |
| Active engagement | ep002_, ac035_, ac036_, sp008_, sp013_, sp014, sp016_, sp018_, dn014_ |
| **AHA-BPS** | |
| Non-frail or pre-frail | mh011_, mh012_, mh013_, gs001_-gs014_, ph048_, br016_ |
| Good cognitive function | cf003_-cf006_, cf007_, cf105_-cf107_, cf012_-cf015_, cf109_- cf 116_ |
| Satisfaction with life | ac012_ |
| No depressive symptoms | mh002_, mh017_, mh014_, mh015_ |
| Social participation | ac035_, ac036_, ac037_, ac038_ |
| Social support | ep002_, sp008_, sp013_, sp014_, sp016_, sp018_, dn014_, MH034_- MH037_ |
| **INDEPENDENT VARIABLES** | |
| Age | dN003_, dn002_ |
| Sex | dn042_ |
| Marital status | dn014_ |
| Educational level | dn010_ |
| Employment status | ep005_ |
| Economic status | co007_ |
| Country of residence | country |
