# Supplementary material for: Prevalence and related factors of Active and Healthy Ageing in Europe according to two models: Results from the Survey of Health, Ageing and Retirement in Europe (SHARE)
Source: PLoS One. 2018 Oct 29;13(10):e0206353. doi: 10.1371/journal.pone.0206353 (PMC6205806; doi:10.1371/journal.pone.0206353)
Supplement: S3 Table — (DOCX) [file pone.0206353.s003.docx]

**S 3 Table. Participant characteristics by country. ***

| Country | AU | BE | CZ | DE | ET | FR | GE | HO | IT | LU | SL | SP | SW | SZ |
| --- | --- | --- | --- | --- | --- | --- | --- | --- | --- | --- | --- | --- | --- | --- |
| **Age** |  |  |  |  |  |  |  |  |  |  |  |  |  |  |
| 50-64 | 53,6 | 54,1 | 53,5 | 52,8 | 52,4 | 53,7 | 52,5 | 55,1 | 50,3 | 58,7 | 58,0 | 53,5 | 48,4 | 52,4 |
| 65-74 | 27,9 | 25,1 | 31,0 | 29,3 | 26,5 | 24,7 | 25,9 | 27,6 | 30,0 | 24,8 | 23,0 | 24,9 | 30,5 | 26,7 |
| 75-84 | 14,0 | 16,3 | 13,0 | 14,2 | 17,4 | 15,7 | 17,4 | 13,6 | 15,8 | 13,7 | 15,2 | 17,1 | 15,8 | 16,5 |
| ≥85 | 4,5 | 4,4 | 2,5 | 3,7 | 3,7 | 5,9 | 4,2 | 3,7 | 3,9 | 2,8 | 3,7 | 4,5 | 5,2 | 4,3 |
| **Sex** |  |  |  |  |  |  |  |  |  |  |  |  |  |  |
| Women | 54,7 | 52,4 | 54,2 | 51,7 | 61,2 | 54,1 | 52,8 | 51,1 | 53,5 | 50,5 | 53,1 | 53,2 | 51,9 | 52,1 |
| **Marital status** |  |  |  |  |  |  |  |  |  |  |  |  |  |  |
| Married | 62,1 | 71,6 | 66,8 | 72,5 | 52,8 | 68,1 | 73,0 | 77,1 | 76,8 | 77,8 | 71,4 | 73,1 | 69,1 | 66,8 |
| Single | 9,6 | 5,9 | 2,9 | 6,3 | 10,4 | 8,1 | 6,1 | 4,6 | 6,4 | 4,5 | 6,7 | 7,6 | 8,7 | 8,4 |
| Divorced/Separated | 13,1 | 11,3 | 15,0 | 11,0 | 16,5 | 10,2 | 9,1 | 7,9 | 3,3 | 8,1 | 5,5 | 5,1 | 13,2 | 14,1 |
| Widowed | 15,2 | 11,2 | 15,4 | 10,2 | 20,2 | 13,6 | 11,9 | 10,3 | 13,5 | 9,7 | 16,4 | 14,2 | 9,1 | 10,7 |
| **Educational level** |  |  |  |  |  |  |  |  |  |  |  |  |  |  |
| Low | 21,9 | 39,2 | 28,4 | 19,5 | 25,2 | 38,2 | 13,3 | 44,7 | 68,6 | 45,2 | 32,4 | 75,3 | 31,3 | 19,2 |
| Moderate | 51,9 | 27,0 | 58,1 | 38,6 | 52,2 | 38,0 | 57,0 | 26,5 | 23,1 | 35,1 | 49,4 | 13,5 | 36,2 | 63,5 |
| High | 26,2 | 33,8 | 13,6 | 41,9 | 22,6 | 23,8 | 29,7 | 28,8 | 8,3 | 19,6 | 18,2 | 11,1 | 32,5 | 17,3 |
| **Employment status** |  |  |  |  |  |  |  |  |  |  |  |  |  |  |
| Retired | 61,1 | 47,1 | 62,6 | 48,5 | 49,7 | 57,0 | 49,5 | 40,6 | 45,7 | 45,8 | 60,2 | 35,5 | 51,9 | 43,7 |
| Employed | 26,5 | 32,5 | 30,0 | 42,8 | 39,4 | 31,3 | 37,1 | 37,9 | 28,8 | 28,0 | 24,5 | 26,3 | 43,8 | 45,2 |
| Other | 12,4 | 20,4 | 7,4 | 8,7 | 10,9 | 11,6 | 13,4 | 21,4 | 25,5 | 26,2 | 15,3 | 38,2 | 4,4 | 11,1 |
| **Economic status^1^** |  |  |  |  |  |  |  |  |  |  |  |  |  |  |
| With difficulty | 16,2 | 24,8 | 44,8 | 10,9 | 58,3 | 28,9 | 22,7 | 16,8 | 57,3 | 17,1 | 58,3 | 46,9 | 12,7 | 12,0 |

*Note: *Percentages are weighted.*

AU: Austria; BE: Belgium; CZ: Czech Republic; DE: Denmark; ES: Estonia; FR: France; GE: Germany; HO: Holland / The Netherlands; IT: Italy; LU: Luxembourg; SL: Slovenia; SP: Spain; SW: Sweden; SZ: Switzerland

^1^ Get to end of month
