# Supplementary material for: Prevalence and related factors of Active and Healthy Ageing in Europe according to two models: Results from the Survey of Health, Ageing and Retirement in Europe (SHARE)
Source: PLoS One. 2018 Oct 29;13(10):e0206353. doi: 10.1371/journal.pone.0206353 (PMC6205806; doi:10.1371/journal.pone.0206353)
Supplement: S4 Table — (DOCX) [file pone.0206353.s004.docx]

**S 4 Table. Prevalence of AHA in both models, by country. ***

|  | **AU** | **BE** | **CZ** | **DE** | **ET** | **FR** | **GE** | **HO** | **IT** | **LU** | **SL** | **SP** | **SW** | **SZ** | **+50** |
| --- | --- | --- | --- | --- | --- | --- | --- | --- | --- | --- | --- | --- | --- | --- | --- |
| **AHA-B model** | | | | | | | | | | | | | | | |
| **D1. Low probability of disease / disability** | **62.4** | **54.4** | **51.9** | **59.7** | **46.6** | **50.0** | **50.0** | **59.2** | **53.7** | **51.7** | **55.3** | **53.9** | **59.1** | **67.9** | **53.0** |
| No disease | 63.9 | 57.3 | 54.1 | 61.5 | 49.6 | 52.3 | 52.0 | 60.7 | 55.9 | 53.1 | 56.7 | 54.9 | 60.8 | 69.9 | 55.0 |
| No disability | 93.2 | 87.9 | 90.7 | 93.2 | 86.7 | 89.3 | 90.8 | 94.2 | 91.4 | 91.4 | 92.9 | 92.1 | 94.2 | 94.8 | 91.1 |
| **D2. High physical & cognitive functioning** | **62.6** | **54.0** | **55.0** | **62.3** | **48.5** | **49.1** | **54.9** | **62.7** | **40.2** | **52.7** | **40.5** | **31.2** | **57.9** | **70.9** | **49.2** |
| High cognitive function | 75.2 | 64.6 | 65.6 | 69.7 | 63.4 | 59.5 | 65.5 | 69.3 | 45.5 | 61.1 | 50.1 | 34.9 | 63.7 | 76.7 | 57.5 |
| High physical function | 77.8 | 79.2 | 77.5 | 85.9 | 70.3 | 77.2 | 78.8 | 86.5 | 77.4 | 79.8 | 71.8 | 75.1 | 87.2 | 90.0 | 78.7 |
| **D3. Active participation** | **54.3** | **59.6** | **57.1** | **75.0** | **55.2** | **61.0** | **60.6** | **70.0** | **49.4** | **57.5** | **46.3** | **44.7** | **69.1** | **65.6** | **57.4** |
| Social participation | 60.9 | 69.5 | 64.7 | 78.4 | 62.0 | 70.7 | 66.9 | 77.3 | 59.3 | 62.6 | 56.8 | 54.1 | 74.6 | 75.2 | 65.7 |
| Social support | 85.6 | 82.9 | 84.6 | 94.9 | 85.6 | 82.8 | 88.1 | 89.1 | 76.8 | 89.5 | 77.8 | 81.4 | 91.0 | 84.9 | 84.0 |
| **TOTAL AHA-B** | **30.8** | **26.1** | **25.9** | **36.8** | **23.2** | **22.8** | **25.5** | **34.6** | **17.7** | **25.4** | **18.0** | **14.1** | **32.2** | **39.5** | **23.5** |
| **AHA-BPS model** | | | | | | | | | | | | | | | |
| **D1. Physical well-being** | **85.4** | **73.9** | **79.7** | **80.6** | **76.4** | **73.9** | **79.5** | **77.4** | **66.1** | **70.1** | **70.3** | **55.5** | **81.6** | **87.0** | **73.1** |
| No frailty | 94.6 | 93.5 | 94.5 | 96.4 | 92.7 | 94.2 | 95.2 | 95.8 | 90.8 | 94.5 | 93.6 | 91.1 | 97.0 | 97.7 | 93.8 |
| High cognition | 89.1 | 78.0 | 82.8 | 83.2 | 81.3 | 77.3 | 82.5 | 80.1 | 70.0 | 73.3 | 73.9 | 57.9 | 83.7 | 88.6 | 76.3 |
| **D2. Mental well-being** | **75.3** | **69.8** | **58.7** | **80.5** | **35.9** | **55.1** | **65.0** | **80.3** | **59.2** | **67.4** | **60.1** | **63.7** | **76.4** | **78.8** | **63.6** |
| Satisfaction with life | 85.7 | 87.1 | 69.7 | 92.9 | 46.2 | 75.1 | 78.3 | 93.5 | 77.6 | 84.7 | 72.1 | 77.5 | 90.4 | 92.3 | 79.2 |
| No depression | 82.8 | 74.7 | 75.4 | 83.6 | 66.1 | 67.3 | 76.8 | 82.8 | 69.4 | 73.9 | 77.7 | 73.1 | 81.1 | 82.8 | 73.8 |
| **D3. Social well-being** | **68.3** | **71.9** | **63.4** | **86.1** | **60.3** | **71.8** | **68.9** | **78.7** | **56.6** | **66.0** | **62.0** | **57.0** | **79.3** | **75.9** | **66.9** |
| Social participation | 70.5 | 77.0 | 69.6 | 88.2 | 64.8 | 76.8 | 73.5 | 84.5 | 62.2 | 70.6 | 66.7 | 62.2 | 83.2 | 80.9 | 71.9 |
| Social support | 95.3 | 91.3 | 87.5 | 97.0 | 89.4 | 91.3 | 91.7 | 92.1 | 86.2 | 91.9 | 88.4 | 89.3 | 94.1 | 92.6 | 90.5 |
| **TOTAL AHA-BPS** | **51.6** | **43.0** | **36.5** | **61.2** | **23.7** | **36.3** | **42.4** | **55.0** | **30.1** | **39.9** | **36.2** | **27.9** | **54.4** | **57.1** | **38.9** |

*Note: *Percentages are weighted.*

AU: Austria; BE: Belgium; CZ: Czech Republic; DE: Denmark; ES: Estonia; FR: France; GE: Germany; HO: Holland / The Netherlands; IT: Italy; LU: Luxembourg; SL: Slovenia; SP: Spain; SW: Sweden; SZ: Switzerland
